# Comparison of fluorescence probes for intracellular sodium imaging in prostate cancer cell lines

**DOI:** 10.1007/s00249-016-1173-7

**Published:** 2016-09-22

**Authors:** Oksana Iamshanova, Pascal Mariot, V’yacheslav Lehen’kyi, Natalia Prevarskaya

**Affiliations:** Inserm U1003, Laboratory of Excellence, Ion Channels Science and Therapeutics, Equipe Labellisée par la Ligue Nationale Contre le Cancer, SIRIC ONCOLille, Université des Sciences et Technologies de Lille, 59656 Villeneuve d’Ascq, France

**Keywords:** Sodium imaging, Fluorescent dye, Prostate cancer cells, SBFI, CoroNa Green, ANG-2

## Abstract

Sodium (Na^+^) ions are known to regulate many signaling pathways involved in both physiological and pathological conditions. In particular, alterations in intracellular concentrations of Na^+^ and corresponding changes in membrane potential are known to be major actors of cancer progression to metastatic phenotype. Though the functionality of Na^+^ channels and the corresponding Na^+^ currents can be investigated using the patch-clamp technique, the latter is rather invasive and a technically difficult method to study intracellular Na^+^ transients compared to Na^+^ fluorescence imaging. Despite the fact that Na^+^ signaling is considered an important controller of cancer progression, only few data using Na^+^ imaging approaches are available so far, suggesting the persisting challenge within the scientific community. In this study, we describe in detail the approach for application of Na^+^ imaging technique to measure intracellular Na^+^ variations in human prostate cancer cells. Accordingly, we used three Na^+^-specific fluorescent dyes–Na^+^-binding benzofuran isophthalate (SBFI), CoroNa™ Green (Corona) and Asante NaTRIUM Green-2 (ANG-2). These dyes have been assessed for optimal loading conditions, dissociation constant and working range after different calibration methods, and intracellular Na^+^ sensitivity, in order to determine which probe can be considered as the most reliable to visualize Na^+^ fluctuations in vitro.

## Introduction

Virtually all living organisms could be unified in their feature to possess sodium (Na^+^) concentration gradient across a plasma membrane. It is reached due to the great difference in ionic composition between interior and exterior regions of the cell. Intracellular Na^+^ concentration ([Na^+^]_i_) is 10–20 times lower than extracellular Na^+^ concentration ([Na^+^]_o_) in every organism. Typically, [Na^+^]_i_ varies around 5–15 mM depending on the cell type in mammals, whereas [Na^+^]_o_ is much higher, around 120–150 mM. Such a concentration gradient is established through the activity of ion channels/transporters and pumps, and is required for maintenance of resting membrane potential. Thus, ion movement across a plasma membrane might initiate electrical excitation as well as local cytoplasmic responses. Therefore, Na^+^ ions have been proposed as a second messenger that could regulate many signaling pathways involved in both physiological and pathological cellular properties (Besson et al. 2015; Roger et al. 2015). In addition to being important in functioning of excitable and non-excitable tissues (e.g., nerve conduction and organogenesis), Na^+^ signaling has also been proposed to regulate cancer cell behaviors (Gillet et al. 2009). Indeed, Na^+^ signaling has been shown to play a significant role in cell polarity, motility, migration, invasiveness, and extracellular matrix remodeling. Importantly, elevated total tissue Na^+^ concentration has been proposed as a highly specific in vivo indicator of malignant lesions in human cancer patients (Jacobs et al. 2011; Ouwerkerk et al. 2003, 2007). Therefore, investigation of Na^+^ signaling dynamics at the cellular level could give us a better understanding of how a variety of carcinogenic processes are being regulated.

Various experimental approaches have been developed to study changes in [Na^+^]_i_. For example, ^23^Na nuclear magnetic resonance spectroscopy offers a reliable method for non-invasive quantification of [Na^+^]_i_ in perfused cancer cells and tissues (Hansen et al. 1993). Flow cytometry also provides feasible [Na^+^]_i_ measurements (Amorino and Fox 1995). However, due to the heterogeneity of cancer cell populations, only large differences could be accurately discriminated by these methods. Functionality of Na^+^ channels, and hence their active Na^+^ currents, can be investigated by usage of the patch-clamp technique (Armstrong 1981; Roden and George 1997). However, all of these options do not comprise the fast and easy-to-perform method to quantify [Na^+^]_i_ variations as Na^+^ fluorescence indicator dyes do. Regarding the fact that Na^+^ signaling is considered as an important controller of cancer progression, it is highly surprising that only a few research groups have published their Na^+^ imaging results using cancer cell lines (Chen et al. 2014; Kline et al. 2000; Roger et al.  2007; Tannous et al. 2009). Indeed, in comparison to well-established calcium (Ca^2+^) imaging, the realization of Na^+^ imaging technique is much less exploited. It is more likely that the main challenge is imposed due to the relatively narrow range of the global [Na^+^]_i_ change (only 10 fold), whereas for Ca^2+^ the differences are much larger (about 10^4^-fold). Thus, for a similar ion flux (Na^+^ versus Ca^2+^), the latter leads to larger relative increases in ion concentration, which makes the detection of Ca^2+^ transients more feasible as compared to Na^+^.

**Fig. 1:**
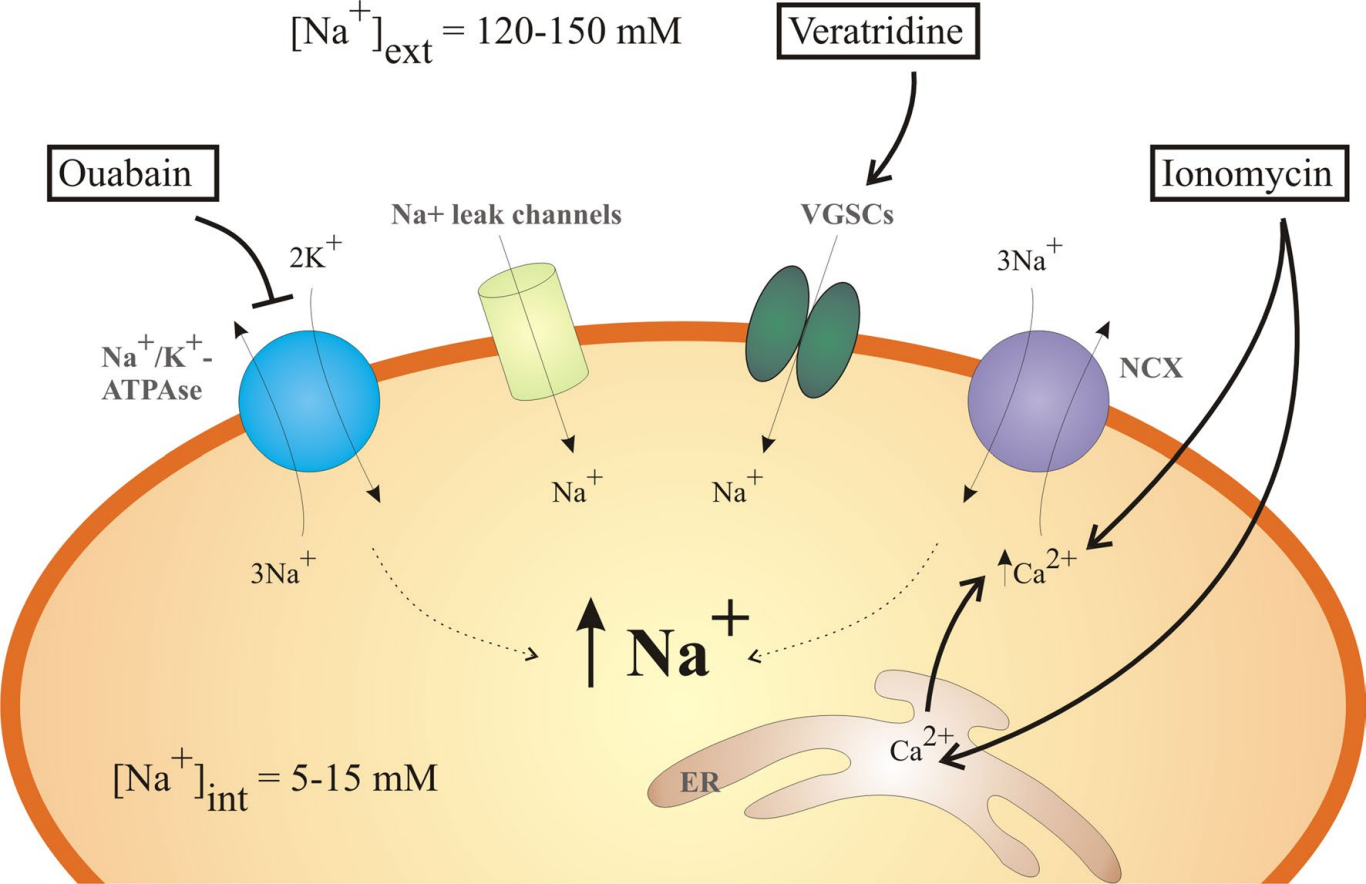
Schematic representation of the molecular pathways used in this study to induce alterations [Na^+^]_i_. Typical Na^+^ concentrations of the mammalian cells are 5–15 mM for cytosol ([Na^+^]_int_) and 120–150 mM for extracellular ([Na^+^]_ext_) medium. [Na^+^]_o_ was switched from 0 to 110 mM for investigating ion diffusion down the electrochemical gradient through Na^+^ leak channels. Ouabain inhibits the activity of Na^+^/K^+^ ATPase and hence restrains constant expulsion of the intracellular Na^+^. The application of voltage-gated Na^+^ channels (VGSCs) opener, veratridine, should also result in [Na^+^]_i_ increase. Ionomycin as an ionophore promotes Ca^2+^ entry through the plasma membrane and the depletion of endoplasmic reticulum (ER) Ca^2+^ stores and hence boosts the activity of Na^+^/Ca^2+^ exchanger (NCX)

In this study, we developed an in vitro Na^+^ imaging approach and compared the suitability of three Na^+^-specific fluorescent dyes–Na^+^-binding benzofuran isophthalate (SBFI), CoroNa™ Green (Corona), and Asante NaTRIUM Green-2 (ANG-2) in prostate cancer cells. The dyes have been evaluated by their various parameters (e.g., loading time, working range, and sensitivity). Modulation of Na^+^ down the diffusion gradient, increase of intracellular Ca^2+^ ([Ca^2+^]_i_), inhibition of sodium/potassium ATPase (Na^+^/K^+^ ATPase), and activation of voltage-gated Na^+^ channels were performed to provide alterations in free [Na^+^]_i_ (Fig. 1).

Overall, this is the first methodological study that provides a detailed description of Na^+^ fluorescence imaging in three human prostate cancer cell lines: DU 145, PC-3, and PC-3M. Moreover, it addresses many technical issues related to the different Na^+^-specific probes and provides a better understanding on their proper application in vitro.

## Materials and methods

### Cell culture

In this study, human prostate cancer cell lines of epithelial origin have been used. DU 145 and PC-3 have been purchased from the American Type Culture Collection (ATCC^®^), whereas—PC-3M have been kindly provided by Prof. Mustafa Djamgoz (Imperial College London, UK). These cancer cell lines derive from various metastatic sites and are relatively classified by their tumorigenic potential as follows: DU 145—issue from brain, moderately metastatic (Stone et al. 1978); PC-3—issue from bone, highly metastatic (Kaighn et al. 1979); and PC-3M—issue from liver, more aggressive derivative of PC-3 (Kozlowski et al. 1984). Cells were routinely cultured at 37 °C in a humidified atmosphere with 5 % CO_2_ in RPMI 1640 medium (Gibco™, Thermo Fischer Scientific) containing a final concentration of 10 % fetal bovine serum (Gibco™) and 2 mM L-glutamine (Gibco™, Thermo Fischer Scientific). Twice a week, cultures were split by treatment with 0.25 % trypsin–EDTA (Gibco™, Thermo Fischer Scientific) for 5 min at 37 °C.

### Solutions

The Hank’s buffer saline solution (HBSS) comprised as follows (mM): NaCl 150, KCl 5, MgCl_2_ 1, CaCl_2_ 2, D-glucose 10, and HEPES 10, adjusted to pH 7.4 with 1 M NaOH. For calibration purposes, two different types of media were used: Na^+^-free solution (Na^+^ was replaced by choline) and a mixture of Na^+^ and potassium (K^+^). Na^+^-free solution contained the following components (in mM): choline chloride 150, MgCl_2_ 1, KCl 5, CaCl_2_ 2, D-glucose 5.6, and HEPES 10, adjusted to pH 7.4 with 1 M KOH. Extracellular solution with high Na^+^ concentration consisted of (mM): NaCl 150, KCl 5, MgCl_2_ 1, CaCl_2_ 2, D-glucose 5.6, and HEPES 10, adjusted to pH 7.4 with 1 M NaOH. Another calibration solution consisted of an ion mixture (in mM) such as [Na^+^ gluconate] + [K^+^ gluconate] = 140, MgCl_2_ 1, CaCl_2_ 2, D-glucose 5.6, and HEPES 10, adjusted to pH 7.4 with 1 M KOH or NaOH. Of note, the final working concentrations of all Na^+^ calibration solutions ranged within 0–120 mM and contained 10 µM gramicidin and monensin. Ouabain-, ionomycin-, and veratridine-containing solutions were prepared in HBSS. All reagents have been purchased from Sigma-Aldrich, unless otherwise specified.

### Sodium fluorescent imaging

All probes were purchased in a form of cell-permeant acetoxymethyl esters: Na^+^-binding benzofuran isophthalate (SBFI) from Interchim (Cat. No. FP-82902B), whereas CoroNa™ Green (CoroNa) and Asante NaTRIUM Green-2 (ANG-2)—from Life Technologies (Cat. No. C36676) and TEFLabs (Cat. No. 3512), accordingly. Dyes were reconstituted in dimethyl sulfoxide (DMSO) and diluted in HBSS containing 0.02 % of Pluronic^®^ F127 to the final concentrations of 7 µM for SBFI, 10 µM for CoroNa, and 5 µM for ANG-2.

Cells were plated onto 30-mm glass coverslips and grown in standard conditions for 3 days. During this time, cells properly attached to the coverslips and had time to divide at least once. In such a way, we reassured that the cells were exhibiting healthy phenotype prior to experimental procedure. The coverslips were then washed with HBSS and loaded with 1 ml of the dye solution as follows: for SBFI–5 h in the incubator at 37 °C, for CoroNa–1 h in the incubator at 37 °C, and for ANG-2–1 h at room temperature. Afterwards, the coverslips were gently rinsed with HBSS, fixed on the imaging platform and left in the appropriate experimental solution. All recordings, including calibrations, were performed at room temperature. About 50 cells per field of view were selected for fluorescence measurements. Background fluorescence was selected as an area containing no cells and subtracted from each data point obtained during experiment.

Fluorescence was excited using an illumination DG4 system (Sutter) fitted with a xenon lamp (300 W). All recordings of Na^+^ fluorescence were acquired using objective 20× in the Superfluor Nikon Eclipse T*i*- series inverted microscope coupled to an EMCCD camera Rolera EM-C^2^ (Qimaging) and processed using Metafluor 7.7.5.0 software. The excitation filters represented the following parameters in terms of wavelength and bandwidth: 340 nm/26 nm and 387 nm/11 nm for SBFI; 482 nm/35 nm for CoroNa and ANG-2. The emission filters were as follows: 510 nm/84 nm for SBFI; 536 nm/40 nm for CoroNa and ANG-2.

### Data analysis and software

For the data that was plotted on a curve and fitted with logistic function, *R*^2^ was calculated as a “least squares” method. Data were analyzed using OriginPro 2015 Beta3 software (1991–2014 OriginLab Corporation). The figures were created using CorelDRAW 11.633 software (2002 Corel Corporation).

## Results

### Basics of sodium imaging and ion-specific indicators

**Fig. 2:**
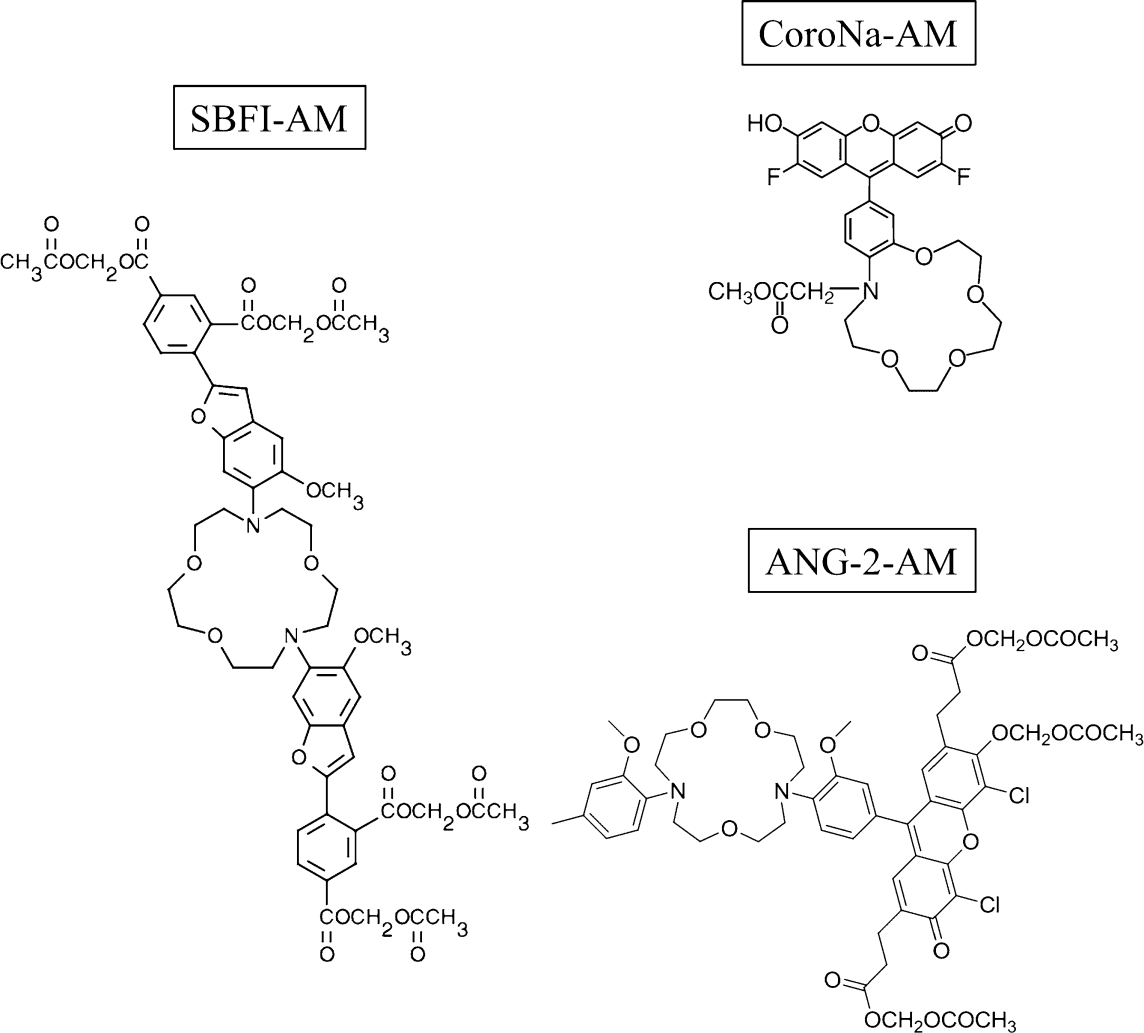
Chemical structures of the different Na^+^ indicator dyes used in this study in the form of acetoxymethyl esters (AM): Na^+^-binding benzofuran isophthalate (SBFI), CoroNa™ Green (CoroNa), and Asante NaTRIUM Green 2 (ANG-2)

The Na^+^ imaging technique is based on the recordings of signals produced by fluorescent Na^+^ indicator dyes. These molecules are typically composed of an ion-binding site and a fluorophore unit (Fig. 2). Thus, the principle is based on dye conformational changes upon Na^+^ binding, which are followed by spectral changes. This can be assessed by exciting dyes’ fluorescence using specific wavelengths and measuring the emitted light, which can be registered and visualized by the software. The choice of commercially available Na^+^ probes is still relatively small, when comparing to the wide range of Ca^2+^ indicator dyes (Rose 2012). Nevertheless, there is still a significant contrast between general chemical properties of the molecules (e.g., fluorescence, ion specificity, affinity). Therefore, these differences have to be accurately considered prior to the dye employment. Importantly, a reliable Na^+^ indicator should be highly specific for Na^+^ over other ions. Hence, Na^+^ binding (or unbinding) should result in a significant change in the fluorescence emission. Furthermore, high dye concentrations and long-term exposure times are not advised for elimination of the possible cytotoxic effects. Thus, emission efficacy of the fluorophore should be as high as possible. To date, Na^+^ indicator dyes are available in the modified form of acetoxymethyl esters. In this form, the passage of the dye across the cell membrane is facilitated. Once inside, the acetoxymethyl groups are removed by cellular esterases and dyes are thus trapped in the cytoplasm.

Despite new developments, the most commonly used Na^+^ indicator dye is still SBFI, first described in 1989 (Minta and Tsien 1989). As a ratiometric dye, it can be excited at two separate wavelengths (340 and 380 nm) and the emitted fluorescence reaches a maximum of intensity at 500 nm. The excitation spectrum is shifted upon Na^+^ binding onto SBFI, the Na^+^-bound SBFI and the Na^+^-free SBFI displaying excitation peaks at 340 and 380 nm, respectively. The ratio of emitted fluorescence obtained at each wavelength (*F*_340nm_/*F*_380nm_) thus directly correlates with [Na^+^]_i_, whereas the SBFI emission spectrum does not depend on changes in [Na^+^]_i_. Of note, the selectivity of SBFI is 18-fold higher towards Na^+^ than towards K^+^.

The properties of CoroNa have been described in detail by earlier studies (Martin et al. 2005; Meier et al. 2006). It is excited by visible light and exhibits an increase in fluorescence emission intensity upon binding of Na^+^ with just a little shift in wavelength. The excitation and emission maxima of CoroNa are at 492 and 516 nm, accordingly, whereas for absorbance it is near 488 nm. The selectivity of CoroNa is 4 times higher to Na^+^ than to K^+^ binding (Martin et al. 2005). Moreover, CoroNa exhibits a relatively high dissociation constant (*K*_d_) making it well suitable for measuring large [Na^+^]_i_ transients (Martin et al. 2005).

Lately, a novel Na^+^ indicator, Asante NaTRIUM Green 1, has been described (Kim et al. 2010; Lamy and Chatton 2011). According to the manufacturer (TEFLabs Inc., Austin, TX, USA), another derivative, ANG-2, shows improved fluorescence properties, but up to now, only few data on this dye have been published (Miyazaki and Ross 2015; Roder and Hille 2014). The excitation and emission maxima of ANG-2 are around 532 and 550 nm, respectively, and Na^+^ binding induces an increase in fluorescence intensity. Noteworthy, ANG dyes select for Na^+^ over K^+^ about 20-fold.

### Loading of the cells with sodium indicator dyes

Within the existing variety of methods for the dye delivery inside of the cell (e.g., microinjection, electroporation, patch-pipette administration, etc.), the usage of esterified compounds is the most practical. The membrane-permeable ester reaches the cytoplasm due to a hydrolytic reaction, and hence no special facilities are required. However, the disadvantage of esters is their ability to accumulate inside intracellular compartments, making the indicator insensitive to the cytosolic levels of Na^+^. It can be avoided by decreasing the loading temperature, which usually implies longer incubation time. Therefore, the loading conditions of each Na^+^ indicator have to be accurately adjusted.

In this study, we optimized loading conditions of esterified SBFI, CoroNa, and ANG-2 indicator dyes when using human prostate cancer cell lines DU 145, PC-3, and PC-3M. The loading conditions were mostly comparable to the manufacturer indications and to other publications (Diarra et al. 2001; Meier et al. 2006; Roder and Hille 2014). It has been reported that the loading time for SBFI may vary depending on the cell type. In our study, the loading times for SBFI and CoroNa were prolonged for 5 and 1 h, respectively. Furthermore, the working concentrations of CoroNa were step-wise increased from 0.5 to 10 µM. Nevertheless, even with high concentrations of CoroNa (10 µM), a gradual fluorescence signal loss was observed, most likely due to the dye leakage out of the cell. Alterations of the loading temperatures did not improve the procedure and hence were not modified. Of note, all three prostate cancer cell lines used in this study represented similar levels of loading within each of the Na^+^-specific dyes under conditions performed (Table 1).

**Table 1:** Comparison of three Na^+^ indicator fluorescent dyes used in this study

Characteristics of Na^+^ indicator dye	SBFI	CoroNa	ANG-2
As indicated by the manufacturer			
Molecular weight (g/mol)	1127.09	657.62	1084
*λ*_ex_ (nm)	340/380	492	517
*λ*_em_ (nm)	505	516	540
Ratiometric	Yes	No	No
Na^+^ selectivity over K^+^	18-fold	4-fold	20-fold
*K*_d_ (absence of K^+^) (mM)	3.8	n/a	20
*K*_d_ (presence of K^+^) (mM)	11.3	80	34
Concentration (µM)	5–10	0.5–10	3–10
Loading time	40 min–4 h	10–45 min	1 h
Loading conditions	37 °C, 5 % CO_2_, humidified	37 °C, 5 % CO_2_, humidified	Room conditions
As used/obtained in this study			
Concentration (µM)	7	10	5
Loading time (h)	5	1	1
*K*_d_ in vitro (choline solution) (mM)	2.4	10.5	3.4
Working range (choline solution) (mM)	0.5–30	1–60	0.5–30
*K*_d_ in vitro (K^+^ mixture) (mM)	20.7	43	32
Working range (K^+^ mixture) (mM)	10–120	10–120	3.75–120

### Calibration of the dyes

When employing Na^+^ indicator dyes, [Na^+^]_i_ is not measured directly. Therefore, calibration is required in order to convert fluorescence signals into defined changes in [Na^+^]_i_. Of note, constructed calibration curves are crucial for estimating multiple working parameters. For example, the dissociation constant K_d_, indicative of the dye affinity for Na^+^, should be within the expected physiological range of [Na^+^]_i_ in the biological sample. Furthermore, efficient measurements of [Na^+^]_i_ changes could be recorded under conditions where the working range of the dye varies between 0.1 to 10 times of its *K*_d_ value. Therefore, these parameters have to be taken into account during the experimental design. Here we describe in vitro calibration procedures of SBFI, CoroNa, and ANG-2 Na^+^ indicator dyes using PC-3 cells that have been sequentially exposed to solutions with different Na^+^ values (ranging from 0 to 120 mM) containing 10 µM monensin and gramicidin.

#### Calibration of ratiometric dye SBFI

**Fig. 3:**
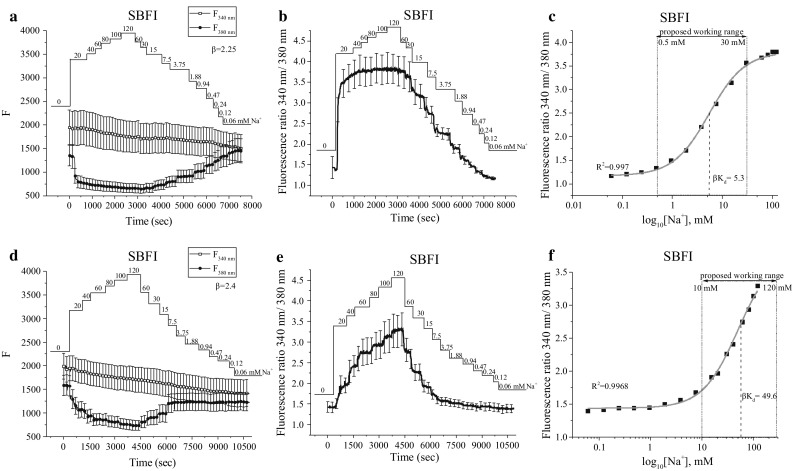
Calibration of SBFI in human prostate cancer PC-3 cells. The real-time [Na^+^]_o_ (0–120 mM) are indicated. Calibrations performed with choline solution are represented in **a**–**c**, whereas with K^+^ solution are shown in **d**–**f**. **a**, **d** Changes in emitted fluorescence intensities during excitation at 340 and 380 nm. **b**, **e** Fluorescence ratio 340 nm/380 nm derived from signals shown in **a** and** d**. In **c** and **f** the data shown is derived from **b** and **e** that have been plotted versus log_10_ of the known Na^+^ concentrations and fitted using a logistic function. Data are represented as mean ± SD

Typical changes in fluorescence emitted during excitation at 340 nm (*F*_340nm_) and 380 nm (*F*_380nm_) have been obtained after calibration and are illustrated as *F*_340nm_ and *F*_380nm_, respectively, in Fig. 3a, d. As expected, *F*_380nm_ inversely correlates with the changes in [Na^+^]_i_, whereas *F*_340nm_ remains essentially unaffected. Accordingly, the fluorescence ratio *F*_340nm_/*F*_380nm_ directly corresponds to the alterations in [Na^+^]_i_ (Fig. 3b, e). Nevertheless, it is still required to convert *F*_340nm_/*F*_380nm_ recordings into [Na^+^] values. Typically for ratiometric dyes as SBFI, it is done by using standard equation (Grynkiewicz et al. 1985):

1 $$[ {\text{Na}}^{ + } ] \;{ = }\;\beta \; \times \;K_{\text{d}} \frac{{R_{\text{n}} \; - \;R_{{{\text{n}}_{ \hbox{min} } }} }}{{R_{{{\text{n}}_{ \hbox{max} } }} \; - \;R_{\text{n}} }},$$

where *β* is *F*_380nm(max)_/*F*_380nm(min)_; *K*_d_ is dissociation constant; *R*_n_ is equal to *F*_340nm_/*F*_380nm_ at each data point recorded; *R*_n(min)_ and *R*_n(max)_ are the fluorescence ratios *F*_340nm_/*F*_380nm_ at the lowest and highest [Na^+^] concentration applied during calibration. Thus, *R*_n_, *R*_n(min)_, and *R*_n(max)_ are known, and *β* can be estimated from the values illustrated in Fig. 3a and d. In order to calculate *K*_d_, the fluorescence ratio *F*_340nm_/*F*_380nm_ was plotted versus log_10_ of known [Na^+^] values used for the calibration (Fig. 3c, f). The derived curve was fitted using a logistic function as follows:

2 $$R_{\text{n}} \;{ = }\;R_{{{\text{n}}_{ \hbox{max} } }} \;{ + }\;\frac{{R_{{{\text{n}}_{ \hbox{min} } }} - R_{{{\text{n}}_{ \hbox{max} } }} }}{{ 1 { + }\;\left( {\frac{{[{\text{Na}}^{ + } ]}}{{K_{\text{d}} }}} \right)^{\text{p}} }},$$

where p is the Hill coefficient, which represents the slope of the linear part of the curve. The EC_50_ was computed and attributed to *β*×*K*_d_ value, from which *K*_d_ was derived. In this study, we used different calibration solutions: in one, Na^+^ was substituted with choline (Fig. 3a–c), whereas in the other it was compensated by K^+^(Fig. 3d–f). Interestingly, the results significantly differed depending on the solutions used for calibration. In the case of choline, *K*_d_ was found to be 2.4 mM, whereas the optimal working range of SBFI was proposed to vary between 0.5 and 30 mM [Na^+^] (Fig. 3c). In the case of the mixture of Na^+^ and K^+^ that was used for calibration, *K*_d_ was found to be 20.7 mM, and the optimal working range lay between 10 and 120 mM [Na^+^] (Fig. 3f).

#### Calibration of non-ratiometric dyes CoroNa and ANG-2

**Fig. 4:**
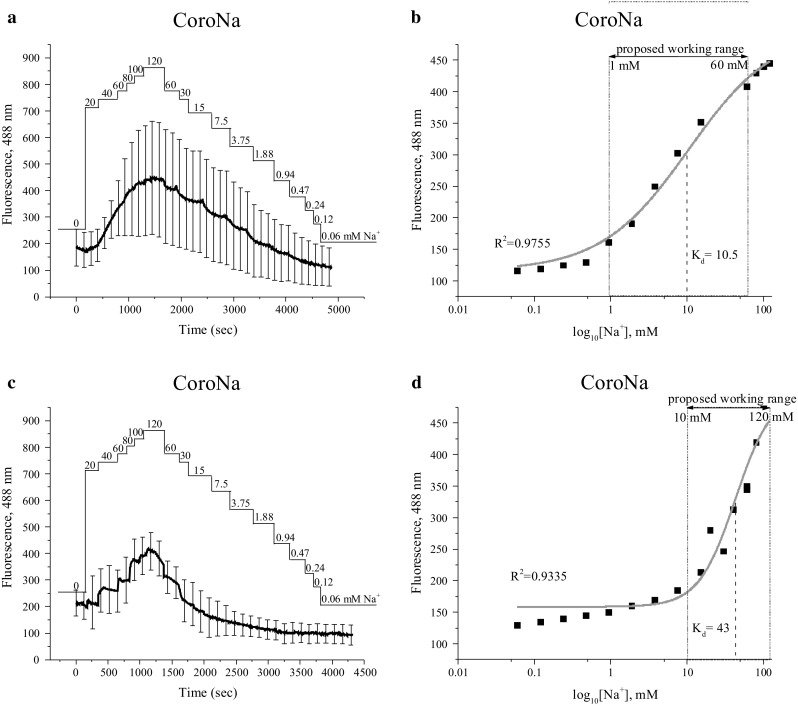
Calibration of CoroNa in human prostate cancer PC-3 cells. The real-time [Na^+^]_o_ (0–120 mM) are indicated. Calibrations performed with choline solution are represented in **a**, **b**, whereas with K^+^ solution in **c**, **d**. **a**, **c** Emitted fluorescence intensities at 488 nm. In **b** and **e** the data shown is derived from **a** and **c** that have been plotted versus log_10_ of the known Na^+^ concentrations and fitted using a logistic function. Data are represented as mean ± SD

**Fig. 5:**
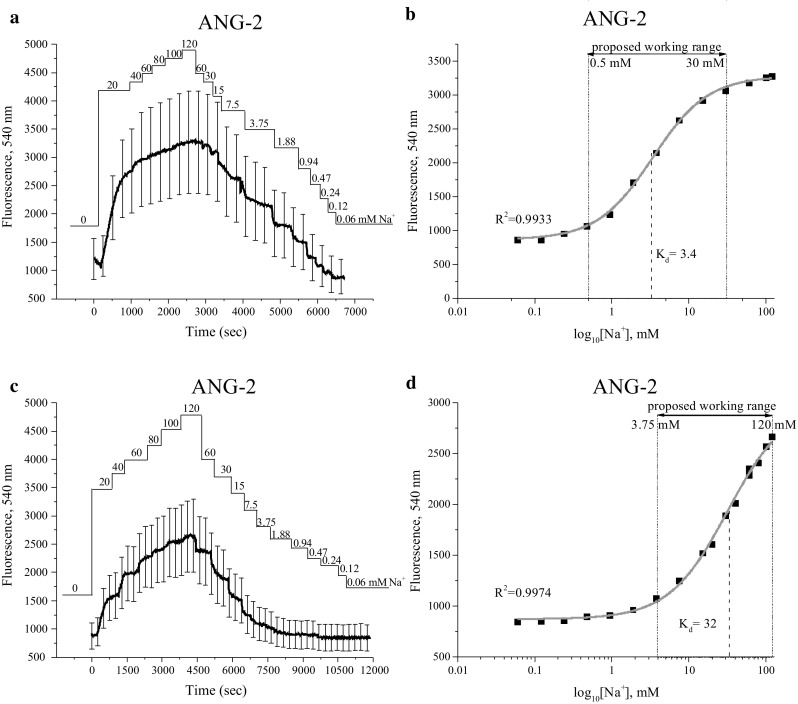
Calibration of ANG-2 in human prostate cancer PC-3 cells. The real-time [Na^+^]_o_ (0–120 mM) are indicated. Calibrations performed with choline solution are represented in **a**, **b**, whereas with K^+^ solution in **c**, **d**. **a**, **c** Emitted fluorescence intensities at 540 nm.  In **b** and **e** the data shown is derived from **a** and **c** that have been plotted versus log_10_ of the known Na^+^ concentrations and fitted using a logistic function. Data are represented as mean ± SD

The calibration of single-wavelength dyes as CoroNa and ANG-2 is relatively simple, and is done with the following equation:

3 $$[ {\text{Na}}^{ + } ] \;{ = }\;K_{\text{d}} \frac{{F - F_{ \hbox{min} } }}{{F_{\text{max }} - F}}$$

where *K*_d_ is dissociation constant, *F* equals the fluorescence signal at each data point recorded, and *F*_min_ and *F*_max_ are the fluorescence signals at the lowest and highest [Na^+^] concentration applied during calibration. The fluorescence intensities obtained after administration of various [Na^+^]_o_ levels are illustrated in Figs. 4a, c and 5a, c. Afterwards, the intensity values were plotted versus log_10_ of [Na^+^] applied (Figs. 4b, d, 5b, d). The logistic function has been used for curve fitting (see Eq. 2), where the value of *K*_d_ has been derived as EC_50_. In choline solution, for CoroNa dye *K*_d_ resulted in 10.5 mM with a working range of between 1 and 60 mM (Fig. 4b), whereas for ANG-2 dye *K*_d_ was 3.4 mM with a working range between 0.5 and 30 mM (Fig. 5b). When using a calibration solution that consisted of a mixture of [Na^+^] and [K^+^], parameters shifted as follows: for CoroNa, *K*_d_ resulted in 43 mM, which lay in the range of 10–120 mM, whereas for ANG-2, *K*_d_ resulted in 32 mM, within the range of 3.75 to 120 mM (Figs. 4d, 5d).

### Response to changes in extracellular sodium concentration

**Fig. 6:**
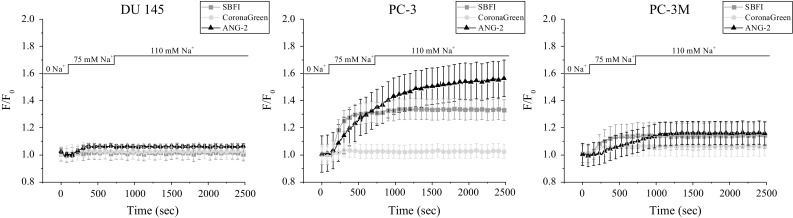
[Na^+^]_i_ levels during Na^+^ switch from 0 to 75 mM and then to 110 mM concentrations. Fluorescence intensities normalized to the initial values (*F*/*F*_0_). Data are represented as mean ± SD

The passive transport of many ions including Na^+^ is carried out across the plasma membrane, down the electrochemical gradient. Thus, in order to investigate the correlation of [Na^+^]_i_ variations as a response to changes in [Na^+^]_o_, we performed a so-called “Na^+^ switch”. First, cells were washed in Na^+^-free media to eliminate residual Na^+^ ion presence. After an initial recording carried out in Na^+^-free solution, [Na^+^]_o_ was switched to 75 mM and then to 110 mM. As indicated in Fig. 6a, PC-3 cells responded to the Na^+^ switch with a 2-fold larger Na^+^ entry than PC-3M, whereas DU 145 exhibited relatively stable levels of [Na^+^]_i_, whatever the [Na^+^]_o_ was. Although Na^+^ diffusion at resting membrane potential is generally accepted as a well-known phenomenon, very little is known about its precise mechanisms. From our results, it is obvious that DU 145 cells are more resistant to [Na^+^]_o_ changes than PC-3 or PC-3M. Thus, it can be suggested that [Na^+^]_i_ in DU 145 cells has reached some limit or saturation point, and hence no more Na^+^ can enter inside. Otherwise, DU145 cells may have efficient Na^+^ buffering mechanisms that allow its rapid extrusion upon Na^+^ entry. In addition, it can be hypothesized that passive Na^+^ transports might be mediated through some ion-specific channels, which are absent/non-functional in DU 145 cells. Nevertheless, these pathways are still scarcely described overall and have to be identified for prostate cancer cells in particular.

### Response to increase in cytosolic calcium concentration

**Fig. 7:**
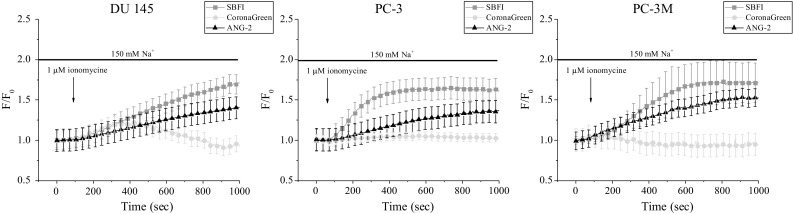
[Na^+^]_i_ levels after cytosolic Ca^2+^ increase induced by an application of 1 µM ionomycin. Fluorescence intensities normalized to the initial values (*F*/*F*_0_). Data are represented as mean ± SD

Ionomycin is an ionophore that is often used as a tool for investigating Ca^2+^-associated mechanisms (Moskowitz and Hruska 1992; Nilsson et al. 1987). Of note, it creates pores in the lipid membrane layer that permit intracellular Ca^2+^ entry. Moreover, after application, ionomycin itself easily penetrates inside of the cell and reaches the endoplasmic reticulum. Consequently, the rise of [Ca^2+^]_i_ is augmented due to endoplasmic reticulum depletion. In this study, we have used ionomycin in order to elevate [Ca^2+^]_i_ (and hence more likely to affect) Na^+^/Ca^2+^ exchanger as the predominant cellular mechanism of Ca^2+^ efflux. Accordingly, in conditions where [Ca^2+^]_i_ is high, the Na^+^/Ca^2+^ exchanger should allow Na^+^ entry, and as a result, [Na^+^]_i_ increases. Here, we have observed a similar rise in [Na^+^]_i_ within all prostate cancer cell lines studied (Fig. 7). This might be explained due to the nature of ionomycin, which is not a cell type-dependent reagent. Hence, its application leads to equal intracellular Ca^2+^ release regardless of the cell line. Consequently, [Ca^2+^]_i_ rise probably affected Na^+^/Ca^2+^ exchanger and thereby provoked comparable changes in [Na^+^]_i_ in all cell lines. Of note, ANG-2 was slightly less sensitive than SBFI, whereas CoroNa exhibited only partial [Na^+^]_i_ fluctuations after ionomycin administration (Fig. 7).

### Response to sodium/potassium pump inhibition

**Fig. 8:**
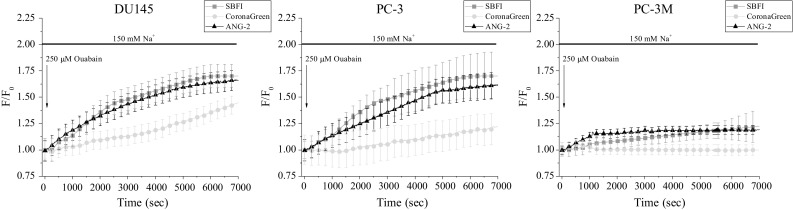
[Na^+^]_i_ levels after Na^+^/K^+^-ATPase inhibition induced by an application of 250 µM ouabain. Fluorescence intensities normalized to the initial values (*F*/*F*_0_). Data are represented as mean ± SD

Na^+^/K^+^ ATPase participates in active ion transport by pumping Na^+^ and K^+^ against their concentration gradients. The enzyme is present in all higher Eukaryotes and hence its functioning has been much investigated. In particular, it has been attributed to the maintenance of the membrane resting potential, cell volume regulation, signal transduction, etc. (Dlouha et al. 1979; Quinton and Tormey 1976; Robinson 1975). Besides controlling intrinsic cellular activities, multiple studies have reported on Na^+^/K^+^ ATPase differential expression in cancer cells and its involvement in cell survival, proliferation, adhesion, migration, and invasiveness (Mijatovic et al. 2008, 2012; Weidemann 2005). Overall, Na^+^/K^+^ ATPase may be considered as one of the most important players of intracellular Na^+^ signaling with its prominent role in cellular (patho)-physiology. Therefore, Na^+^ imaging is an essential technique for visualization of Na^+^/K^+^ ATPase functioning in vitro. In the present study, the activity of Na^+^/K^+^ ATPase was examined by the application of a pump-specific inhibitor, ouabain, at a concentration of 250 µM. As expected, all cell lines tested responded with a progressive accumulation of [Na^+^]_i_ (Fig. 8). Interestingly, DU 145 and PC-3 cells exhibited higher [Na^+^]_i_ elevations than PC-3M cells (Fig. 8). This could imply various expression levels of this enzyme among different prostate cancer cell lines. Noteworthy, all three Na^+^ indicator dyes were able to detect [Na^+^]_i_ increase in DU 145 and PC-3 cells (Fig. 8). However, CoroNa exhibited lower sensitivity for [Na^+^]_i_ elevation in these cells and was not sufficiently effective to uncover lower response in PC-3M cells (Fig. 8).

### Response to voltage-gated sodium channel opener

**Fig. 9:**
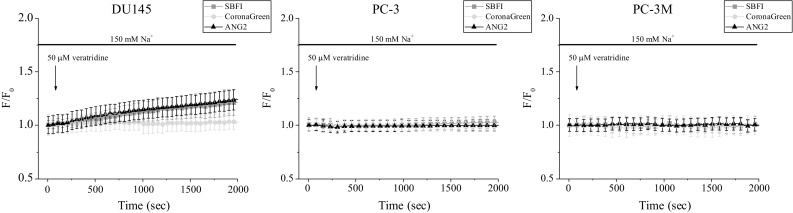
[Na^+^]_i_ levels after voltage-gated Na^+^ channels opening induced by an application of 50 µM veratridine. Fluorescence intensities normalized to the initial values (*F*/*F*_0_). Data are represented as mean ± SD

Voltage-gated Na^+^ channels are known for their involvement in allowing action potential triggering and propagation in nerve and muscle tissues (Denac et al. 2000). However, there is growing evidence on the implementation of these channels in “non-excitable” epithelial cells (Barshack et al. 2008; Gao et al.  2009). Especially, voltage-gated Na^+^ channels have been found upregulated in many carcinomas, and in particular associated with their metastatic potential (Patel and Brackenbury 2015; Roger et al. 2015). Therefore, in our study we have used DU 145, PC-3, and PC-3M cells, which are considered as a highly aggressive model of human prostate cancer. Indeed, these cell lines have been reported to express voltage-gated Na^+^ channels (Nakajima et al. 2009; Shan et al.  2014). Thus, we have investigated their functioning using a channel-specific opener, veratridine. Surprisingly, as measured using SBFI and ANG-2, voltage-gated Na^+^ channel opener provoked a minor [Na^+^]_i_ increase in DU 145 cells and had no effect on PC-3 and PC-3M (Fig. 9a, b). Partially, it can be explained due to the lack of the effect of veratridine itself, which has already been reported for rat prostate cancer cells (Fraser et al. 2000). In addition, though voltage-gated Na^+^ channels have been shown to be expressed in prostate cancer cells (Suy et al. 2012), the resting membrane potential may not be compatible with their activity. In that case, the channels may not permeate significant Na^+^ entry into the cells, which could also explain the lack of the effect of veratridine. We also cannot exclude that the probes used in this study are not sensitive enough to detect these variations of [Na^+^]_i_. Of note, the value of veratridine as a chemical activator of voltage-gated Na^+^ channels has been undermined since it was shown to have only a slight influence on the tetrodotoxin-resistant channel in non-voltage-clamped cells (Farrag et al. 2008). Overall, it has been previously suggested that veratridine may interact with other ion channels and hence its mode of action may be rather complex (Fraser et al. 2003; Romey and Lazdunski 1982; Verheugen et al. 1994).

## Discussion

In vitro Na^+^ imaging has already been reported in various types of cells (Baquero and Gilbertson 2011; Komlosi et al. 2003; Lahn et al. 2011; Miyazaki and Ross 2015). However, only few data have been reported on its particular application in cancer cells (Chen et al. 2014; Kline et al. 2000; Roger et al.  2007; Tannous et al. 2009). Indeed, due to the relatively small differences of Na^+^ concentrations (as compared to the Ca^2+^ gradient) between intra- and extracellular compartments (around 10 times), the detection of the signal is a rather challenging procedure. Therefore, it is not surprising that only several Na^+^ indicator dyes, such as SBFI, CoroNa, and ANG-2, are commercially available up to date.

Since technical limitations may become decisive for a whole experimental design, it is important to take into account some major aspects of the Na^+^ dyes use when performing Na^+^ imaging. For example, the employment of such ratiometric dye as SBFI eliminates experimental variables as cell thickness or size, photobleaching, and dye concentration. However, it is sensitive to changes in intracellular pH (Diarra et al. 2001). Therefore, Na^+^ recordings involving significant pH alterations should be reconsidered using an alternative Na^+^ indicator dye. However, a ratiometric dye enables direct comparison between different experiments and quantitative measurement of signals, and, if precise [Na^+^]_i_ estimation is required while using single-excitation wavelength dye, the calibration procedure has to be performed on each sample tested. The employment of the latter becomes markedly inconvenient for Na^+^ imaging in multiple samples, which is often required to reach statistical significance, especially when the response is transient and/or of a low magnitude.

Importantly, results of calibration were remarkably different depending on the solutions utilized. For example, when Na^+^ was substituted by choline, the saturation point for SBFI and ANG-2 dyes was already reached at around 20–40 mM [Na^+^]_o_ with the proposed working range within 0.5 and 30 mM. In the case when Na^+^ concentration was compensated by K^+^, the saturation point as well as the working range were shifted towards higher [Na^+^]_o_. It is possible that due to electrochemical gradient, the endogenous high [K^+^]_i_ creates some limitation for other cations to enter the cell. Therefore, in this case, performing calibration when increasing [K^+^]_o_ would affect K^+^ exit out of the cell and hence impede cellular capacity for Na^+^ entry.

Interestingly, the calibration of all three Na^+^ indicator dyes has shown that CoroNa is more suitable for detecting [Na^+^]_i_ variations in a wider range of concentrations. Its working range and *K*_d_ are at least twice as large as SBFI or ANG-2. Indeed, CoroNa has been previously reported to discriminate substantial [Na^+^]_i_ changes at high background [Na^+^] (Meier et al. 2006). SBFI, CoroNa, and ANG-2 are not toxic per se and there have not been any pharmacological effects of the indicators demonstrated yet. However, short exposure times and low indicator concentrations are favorable for minimizing cellular stress. In this case, the disadvantage of SBFI is its slow cell permeability, even after addition of special dispersion agent Pluronic^®^ F127. For example, in this study, it took 5 h to charge human prostate cancer cells DU 145, PC-3, and PC-3M. Nevertheless, it has been reported that SBFI loading strongly depends on the cell type and hence it is possible that for some other in vitro models this issue will not occur (Diarra et al. 2001; Minta and Tsien 1989).

This study has been conducted to compare three different Na^+^-specific fluorescent dyes and their feasibility for in vitro Na^+^ imaging (Table 1). Once the working parameters were derived from the calibration curves, it was necessary to examine the dyes suitability within experimental conditions. Therefore, changes in free [Na^+^]_i_ have been initiated via diverse pathways—ubiquitous and cell type-specific. For example, various types of human cancer cells, including prostate, have been reported to express voltage-gated Na^+^ channels (Fraser et al. 2014; Shan et al. 2014). Thus, application of the Na^+^ channel activator veratridine would lead to [Na^+^]_i_ increase only in cells where functional Na^+^ channels are expressed on the plasma membrane. However, in this study veratridine application did not exhibit large Na^+^ entry, more likely due to its complex mode of action (Fraser et al. 2003). Na^+^/K^+^ ATPase, a protein that transports three Na^+^ out of the cell against two K^+^ inside, is found in the plasma membrane of all animal cells (Dlouha et al. 1979; Quinton and Tormey 1976; Robinson 1975). Accordingly, the application of ouabain, a specific inhibitor of Na^+^/K^+^ ATPase, resulted in omnipresent [Na^+^]_i_ increase. Similarly, ionomycin is an ionophore that acts on any kind of cell where it enables Ca^2+^ entry through the plasma membrane and its release from the endoplasmic reticulum (Moskowitz and Hruska 1992; Nilsson et al. 1987). Possibly the elevated [Ca^2+^]_i_ affected the activity of Na^+^/Ca^2+^ exchanger that imports Na^+^ and hence increased [Na^+^]_i_. In addition, Na^+^ entry can be provided via ion diffusion down the electrochemical gradient through Na^+^-specific leak channels (Pajor and Wright 1992; Parent et al. 1992; Wright et al. 1992). In this study, the function of Na^+^-leak channels has been investigated due to the alteration of [Na^+^]_o_. Interestingly, the changes of [Na^+^]_o_ induced more rapid alterations of SBFI signal than ANG-2. Therefore, we suggest that ANG-2 is a slightly less dynamic probe than SBFI.

Of note, CoroNa has exhibited lower signal intensities than SBFI or ANG-2 throughout all experiments. This could be interpreted due to the elevated *K*_d_ and working range of CoroNa, which means that the dye may work more accurately for higher [Na^+^] alterations. Another explanation could be attributed to the leakage of CoroNa out of the cells with time that has been observed in this study. Indeed, previous reports indicate that CoroNa may be lost quickly from intracellular medium due to its relatively small size. This can be alleviated with a constant delivery of the dye through a patch-pipette during whole-cell path clamp recordings, which enables stable intracellular CoroNa concentrations (Meier et al. 2006). In a promising novel approach, Lamy and coworkers encapsulated CoroNa Green in a PAMAM dendrimer-nanocontainer to counteract permanent dye extrusion (Lamy et al. 2012). SBFI and ANG-2 have exhibited comparable levels of signal intensities after various [Na^+^]_i_ alterations. However, it has to be kept in mind that Na^+^ imaging conducted with the SBFI dye enables a reliable and quantitative determination of the actual [Na^+^]_i_, whereas loading conditions for ANG-2 are more practical.

In conclusion, this study presents in vitro Na^+^ imaging in three human prostate cancer cell lines. According to our data, CoroNa is not suitable for the detection of relatively low [Na^+^]_i_ changes in the prostate cancer cell model studied here, whereas SBFI and ANG-2 can provide reliable recordings.
